# MERS Coronavirus in Dromedary Camel Herd, Saudi Arabia

**DOI:** 10.3201/eid2007.140571

**Published:** 2014-07

**Authors:** Maged G. Hemida, Daniel K.W. Chu, Leo L.M. Poon, Ranawaka A.P.M. Perera, Mohammad A. Alhammadi, Hoi-yee Ng, Lewis Y. Siu, Yi Guan, Abdelmohsen Alnaeem, Malik Peiris

**Affiliations:** King Faisal University, Al Hofuf, Saudi Arabia (M.G. Hemida, M.A. Alhammadi, A. Alnaeem);; Kafrelsheikh University, Kafr Elsheikh, Egypt (M.G. Hemida);; The University of Hong Kong, Hong Kong, China (D.K.W. Chu, L.L.M. Poon, R.A.P.M. Perera, H.-y. Ng, L.Y. Siu, Y. Guan, M. Peiris)

**Keywords:** zoonosis, MERS, genomics, phylogeny, mutation, transmission, viruses, Saudi Arabia, Middle East respiratory syndrome coronavirus

## Abstract

A prospective study of a dromedary camel herd during the 2013–14 calving season showed Middle East respiratory syndrome coronavirus infection of calves and adults. Virus was isolated from the nose and feces but more frequently from the nose. Preexisting neutralizing antibody did not appear to protect against infection.

Ongoing transmission of Middle East respiratory syndrome coronavirus (MERS-CoV) to humans underscores the need to understand the animal sources of zoonotic infection (*1*,*2*). MERS-CoV RNA has been detected in dromedary camels (*3*,*4*), and dromedary infection precedes human infection (*5*). We conducted a prospective study in dromedary herds in Al-Hasa, Saudi Arabia, through the peak calving season (December 2013–February 2014) to document virologic features of MERS-CoV infection in these animals.

## The Study

We studied dromedaries at 2 farms in Al-Hasa, 4–5 km apart. Farm A had 70 animals; 4 were 1 month of age, 8 were ≈1 year of age, and the rest were adults (>2 years of age). The herd did not go to pasture in the desert (“zero-grazing”; type of grazing may influence types of potential exposures). The animals were sampled on 5 occasions during November 2013–February 2014. Farm B (“semi–zero-grazing”) had 17 adults and 3 calves; its herd was sampled in February 2014. Nasal, oral, or rectal swab samples and blood samples were collected (Table 1; Technical Appendix Table). Swab and serum samples were stored frozen at −80°C until testing.

**Table 1:** RT-PCR of dromedary camel samples for MERS-CoV, Al-Hasa, Saudi Arabia*

Farm, sampling date	Age†/no. sampled	No. specimens positive/no. tested		
Nasal	Oral	Fecal
Farm A				
2013 Nov 30	Calf, 0	ND	ND	ND
	Adult, 4	1/1	0/2	0/4
2013 Dec 4	Calf, 9	ND	0/9	0/7
	Adult, 2	ND	0/2	0/2
2013 Dec 30	Calf, 8	7/8	0/1	0/6
	Adult, 3	1/3‡	0	1/3‡
2014 Feb 14	Calf, 7	0/7	ND	0/7
	Adult, 2	0/2	ND	0/2
Farm B: 2014 Feb 11	Calf, 3	0/3	ND	0/3
	Adult, 3	0/3	ND	0/3

*Data on individual dromedaries are provided in online Technical Appendix Table, wwwnc.cdc.gov/EID/article/20/7/14-0571-Techapp1.pdf. RT-PCR, reverse transcription PCR; MERS-CoV, Middle East respiratory syndrome coronavirus; ND, not done. †Adults are 6–14 y of age; calves are 40 d to 2 y of age. ‡Two different dromedaries were positive in nasal and fecal swabs.

Hydrolysis probe–based real-time reverse transcription PCR (RT-PCR) targeting MERS-CoV upstream of E (UpE) and open reading frame (ORF) 1a genes and a broad-range RT-PCR reacting across the CoV family to detect other CoVs were used as described (*4*). Specimens initially positive for MERS-CoV were re-extracted and retested to confirm the positive results.

The full genome of MERS-CoV was obtained directly from the clinical specimens with 3–4 times coverage by sequencing PCR amplicons with overlapping sequence reads and sequence assembly (*4*). Dromedary MERS-CoV full genomes obtained in this study (GenBank accession nos. KJ650295–KJ650297) were aligned with human MERS-CoV genomes retrieved from GenBank. We constructed full-genome phylogenies using MEGA5 with neighbor-joining and bootstrap resampling of 500 replicates (*6*). Virus isolation was attempted in Vero E6 cells. We tested serum samples for neutralizing antibody titers using a validated MERS-CoV spike pseudoparticle neutralization test (*7*) (online Technical Appendix).

At farm A, we detected MERS-CoV in 1 of 4 dromedaries sampled on November 30, none of 11 sampled on December 4, nine of 11 sampled on December 30, and none of 9 sampled on February 14 (Table 1). Of the 10 dromedaries that tested positive for MERS-CoV, 9 had parallel nasal and fecal specimens tested, with virus detected in the nasal swab specimens from 8 and the fecal specimen from 1. At the December 30 sampling, 7 of 8 calves and 2 of 3 adults tested positive for MERS-CoV, indicating that when MERS-CoV circulates on a farm, both calves and adults can be infected (Technical Appendix Table). Because all 12 adults with serum collected before December 30 were seropositive (titers >320), it is likely, though not certain, that the MERS-CoV infections in the 2 adults (nos. 21, 19Dam) sampled on December 30 were reinfections, as has been reported for other CoVs (*8*). The seronegative 1-year-old calves, nos. 13 and 14, had the highest nasal viral loads (UpE assay 1.3 × 10^8^ to 1.78 × 10^8^/mL specimen), and a 2-week-old calf, no. 22, with (presumably passively acquired) titers of 1,280 became infected but had a much lower viral load. Overall, these data suggest that prior infection or passively acquired maternal antibody might not provide complete protection from infection (Technical Appendix Table).

Four MERS-CoV–positive calves had mild respiratory signs (cough, sneezing, respiratory discharge), abnormally elevated body temperature, and loss of appetite at the December 30 sampling, which resolved over a few days. Three calves from which paired serum samples were available (Table 2; nos. 13, 15, 17) demonstrated >4-fold rising antibody titers to MERS-CoV. Calf no. 13 (1 year of age) had a high viral load and was seronegative at the first MERS-CoV–positive result (indicating that it had been recently infected) but was MERS-CoV RNA negative 6 weeks later, suggesting that virus shedding is not prolonged. We did not detect virus RNA by RT-PCR in the 3 acute-phase serum samples from infected dromedaries (nos. 1, 16, 17), suggesting that acute infection is not associated with prolonged viremia. Dromedaries from farm B were sampled once on February 11; all results were negative.

**Table 2:** Longitudinal sampling of MERS-CoV–positive dromedary camel calves on farm A, Al-Hasa, Saudi Arabia*

Calf no.	Sample collection date	Sex/age	RT-PCR result	Titer
13	2013 Dec 30	F/1 y	Positive	<20
	2014 Feb 14	F/1 y	Negative	640
15	2013 Dec 30	F/1 y	Positive	20
	2014 Feb 14	F/1 y	Negative	160
17	2013 Dec 30	F/40 d	Positive	80
	2014 Feb 14	F/3 mo	Negative	1,280
19	2013 Dec 30	F/1 y	Positive	NA
	2014 Feb 14	F/1 y	Negative	320
*MERS-CoV, Middle East respiratory syndrome coronavirus; RT-PCR, reverse transcription PCR				

The full genomes of MERS-CoV sequenced directly from a nasal swab specimen collected on November 30 were identical to those from a nasal swab specimen and a fecal specimen collected on December 30. In addition, the complete spike gene was sequenced from 4 other MERS-CoV–positive nasal swab specimens, and these spike genes were genetically identical.

Virus isolation in Vero E6 cells was attempted from 7 positive nasal swab and fecal specimens that had >10^6^ copies/mL in the original sample in the UpE RT-PCR. Viruses were isolated from 2 nasal swab (nos. 13, 14) and 1 fecal swab (no. 19Dam) specimens collected on December 30; these were the specimens with high numbers of MERS-CoV copies (9.27 × 10^7^ to 1.78 × 10^8^ copies/mL). The full-genome sequence of 1 virus culture isolate was obtained in parallel with that of the original virus in the original clinical specimen. We observed 3 nucleotide changes in ORF1b, spike, and membrane protein genes in the isolates after 2 passages in Vero E6 cells, of which 2 were nonsynonymous, leading to changes in spike (S1251F) and membrane proteins (T8I). This finding highlights the importance of sequencing the viral genome directly from clinical specimens.

MERS-CoVs circulating in dromedaries on farm A during a 1-month period were genetically identical over the full 30,100-nt genome in 3 viruses and the spike protein of 4 more viruses, giving a mutation rate of 0 nt substitutions per site per day (95% credible interval 0 to 2.7 × 10^−6^). The estimated mutation rate for epidemiologically unlinked human MERS-CoV was 3.1 × 10^−6^ (95% CI 2.4 × 10^−6^ to 3.8 × 10^−6^) (*9*).

## Conclusions

The unusual genetic stability of MERS-CoV in dromedaries, taken together with its high seroprevalence (*7*,*10**–**13*), raises the hypothesis that dromedaries might be the natural host for this virus. Further longitudinal studies of MERS-CoVs in dromedaries are needed to confirm this hypothesis.

Genome organization of the dromedary MERS-CoV detected in this study was identical to that of the virus in humans. The virus strains clustered phylogenetically within clade B (*9*) and were most closely related to the strain MERS-CoV_FRA/UAE and to MERS-CoV detected in Buraidah (Saudi Arabia) and Al-Hasa (Figure). The farm is ≈300 km from United Arab Emirates and 600 km from Buraidah. Dromedaries move between Al-Hasa and Buraidah and, more limitedly, between Al-Hasa and United Arab Emirates.

**Figure:**
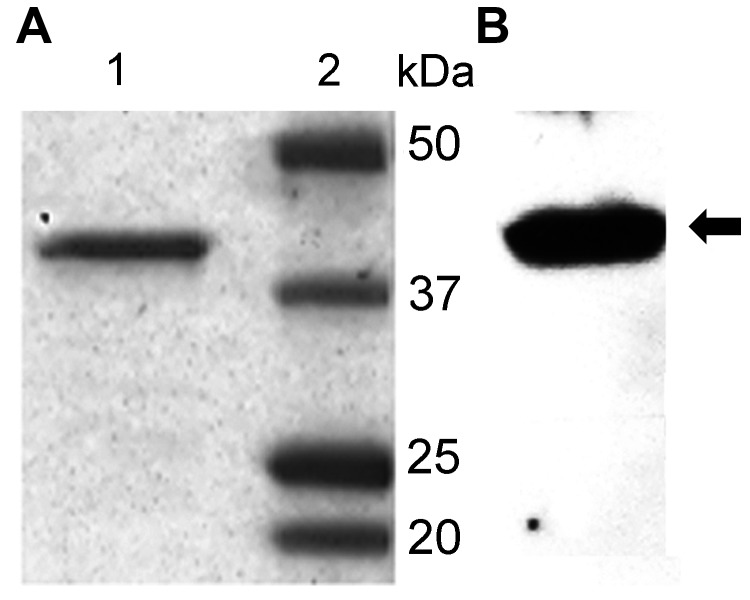
Phylogenetic tree of Middle East respiratory syndrome coronavirus (MERS-CoV) full genomes (29,901 nt after trimming the ends) or near–full genomes from humans and dromedary camels. The tree was constructed by using neighbor-joining methods with bootstrap resampling of 500 replicates. The most divergent MERS-CoV, Egypt NRCE-HKU205, was used as outgroup. Bold type indicates camel MERS-CoV genomes from this study. GenBank accession numbers of genome sequences included in this study are KJ477102, KF600652, KF600630, KF600651, KF186567, KF600627, KF186564, KF600634, KF600632, KF600644, KF600647, KF600645, KF186565, KF186566, KF745068, KF600620, KF600612, KC667074, KC164505, KF192507, KF600613, KF600628, KF961222, KF961221, KC776174, and JX869059. Scale bar indicates nucleotide substitutions per site.

The full-genome sequence of MERS-CoV from dromedaries in this study is 99.9% similar to genomes of human clade B MERS-CoV. The spike gene is the major determinant for virus host specificity. In comparison with other publically available human MERS-CoV sequences, we found 6-nt mutations in the spike gene unique to these dromedary viruses. Of these, 3 (S457G, L773F, and V810I) were nonsynonymous. These amino acid changes are located outside the binding interface between MERS-CoV spike protein and human DPP4 receptor, suggesting these amino acid differences are unlikely to affect receptor binding. Thus, these dromedary viruses may retain capacity to infect humans, as Chu et al. suggested for dromedary MERS-CoV in Egypt (*4*).

MERS-CoV may be isolated from nasal swab specimens and feces, indicating that both could be possible sources of virus transmission to humans and other animals, but virus detection rates were higher in nasal swab specimens. Our preliminary data suggest that preexisting MERS-CoV antibody might not completely protect against re-infection; however, this question needs more investigation.

Technical Appendix Testing of dromedary camels by reverse transcription PCR and serologic testing for Middle East respiratory syndrome coronavirus, Al-Hasa, Saudi Arabia; and detailed methods used in this study
