# Supplementary material for: MERS Coronavirus in Dromedary Camel Herd, Saudi Arabia
Source: Emerg Infect Dis. 2014 Jul;20(7):1231–4. doi: 10.3201/eid2007.140571 (PMC4073860; doi:10.3201/eid2007.140571)
Supplement: Technical Appendix — Testing of dromedary camels by reverse transcription PCR and serologic testing for Middle East respiratory syndrome coronavirus, Al-Hasa, Saudi Arabia; and detailed methods used in this study [file 14-0571-Techapp-s1.pdf]

Article DOI: http://dx.doi.org/10.3201/eid2007.140571

Middle East Respiratory Syndrome Coronavirus in Dromedary Camel Herd, Saudi Arabia

Technical Appendix

Technical Appendix Table. Testing of dromedary camels by RT-PCR and serologic testing for MERS-CoV, Al-Hasa, Saudi Arabia*

Farm, sampling date	Camel no.	Calf/Adult†	Age	RT-PCR result: Nasal sample	RT-PCR result: Oral sample	RT-PCR result: Fecal sample	Copy/mL‡	Antibody titers§
Farm A								
2013 Nov 30	1	Adult	13 y	Pos¶	Neg	Neg	2.61×10^7	>5,120
	2	Adult	12 y	–	–	Neg		>5,120
	3	Adult	10 y	–	Neg	Neg		>5,120
	4	Adult	14 y	–	–	Neg		>5,120
2013 Dec 4	5	Adult	8 y	–	Neg	Neg		640
	6	Adult	9 y	–	Neg	Neg		2,560
	2	Adult	10 y	–	Neg	–		>5,120
	7Calf	Calf	1–2 y	–	Neg	Neg		1,280
	7Dam	Adult	9.5 y	–	Neg	–		2,560
	8	Adult	7 y	–	Neg	Neg		1,280
	9	Adult	6 y	–	Neg	Neg		1,280
	10	Adult	8 y	–	Neg	Neg		640
	11Calf	Calf	1–2 y	–	Neg	Neg		–
	11Dam	Adult	–	–	Neg	Neg		–
	12	Adult	12 y	–	Neg	Neg		320
2013 Dec 30	13	Calf	1 y	Pos¶#	–	–	1.30×10^8	<20
	14	Calf	1 y	Pos#	–	Neg	1.78×10^8	<20
	15	Calf	1 y	Pos	–	Neg	6.07×10^6	20
	16	Calf	1 y	Pos	–	Neg	3.78×10^7	>5,120
	17	Calf	40 d	Pos	–	Neg	4.86×10^4	80
	18	Calf	40 d	Neg	Neg	–		–
	19Calf	Calf	1 y	Pos	–	Neg	2.41×10^7	–
	19Dam	Adult	–	Neg	–	Pos¶#	9.27×10^7	–
	20	Adult	8 y	Neg	–	Neg		>5,120
	21	Adult	7 y	Pos	–	Neg	3.31×10^3	320
	22	Calf	2 wk	Pos	–	Neg	3.38×10^3	1,280
2014 Feb 14	26	Calf	9 mo	Neg	–	Neg		>5,120
	13	Calf	1 y	Neg	–	Neg		640
	27	Calf	1 0 mo	Neg	–	Neg		40
	15	Calf	1 y	Neg	–	Neg		160
	17	Calf	3 mo	Neg	–	Neg		1,280
	11Dam	Adult	12 y	Neg	–	Neg		1,280
	19Calf	Calf	1 y	Neg	–	Neg		320
	28Calf	Calf	3 mo	Neg	–	Neg		20
	28Dam	Adult	10 y	Neg	–	Neg		1,280
Farm B, 2014 Feb 11	23Calf	Calf	2.5 mo	Neg	–	Neg		–
	23Dam	Adult	7 y	Neg	–	Neg		>5120
	24Calf	Calf	2 mo	Neg	–	Neg		–
	24Dam	Adult	6 y	Neg	–	Neg		1,280
	25Calf	Calf	2 mo	Neg	–	Neg		–
	25Dam	Adult	6 y	Neg	–	Neg		640

*RT-PCR, reverse transcription PCR; MERS-CoV, Middle East respiratory syndrome coronavirus; Pos, positive; Neg, negative; –, specimen not collected or age information not available.
†Calf defined as dromedary camel <2 y of age; adult defined as dromedary camel ≥2 y of age.
‡Data deduced from the upstream of E assay.
§Pseudotype neutralization antibody titers.
¶Full genome sequenced.
#Virus isolated.

Methods

PCR

Hydrolysis probe-based real-time PCRs targeting upstream of E gene (UpE) and open reading frame (ORF) 1a were used as recommended by the World Health Organization (WHO). We also tested each specimen with broad-range reverse transcription PCR (RT-PCR) conserved across the coronavirus family to detect other coronaviruses. The nucleic acid extraction methods, primers, and PCR testing protocols used have been described (*1*).

In brief, nucleic acid was extracted from 140-μL aliquots of swab supernatants using QIAamp Viral RNA Minikit (QIAGEN, Hilden, Germany) following the procedure recommended by the manufacturer. Viral RNA was eluted in 60 μL elution buffer provided in the kit, and 12 μL was used for preparing a 20-μL reverse transcription reaction with Superscript III reverse transcriptase (Life Technologies, Carlsbad, CA, USA). Complementary DNA (cDNA) produced was subjected to the detection of Middle East respiratory syndrome coronavirus (MERS-CoV) by using probe-based real-time PCRs targeting UpE and ORF1a as recommended by WHO. For the upE qPCR, a 15-μL reaction was set up containing 1 μM of each of the forward (5′-GCAACGCGCGATTCAGTT-3′) and reverse (5′-GCCTCTACACGGGACCCATA-3′) primers, 0.5 μM probe (5′6-FAM/CTCTTCACATAATCGCCCCGAGCTCG/3′6-TAMSp), and 2 μL cDNA in 1× reaction buffer (Takara, Kyoto, Japan). The reaction was carried out in ViiA 7 real-time PCR system (Life Technologies) with 40 cycles of amplification. For the ORF1a quantitative PCR, the reaction was set up as above except with forward primer (5′-CCACTACTCCCATTTCGTCAG-3′), reverse primer (5′-CAGTATGTGTAGTGCGCATATAAGCA-3′), and probe (5′6-FAM/TTGCAAATTGGCTTGCCCCCACT/3′6-TAMSp) targeting the ORF1a gene. Cycle threshold was generated by the ViiA7 system automatically with default settings.

The full genome of MERS-CoV in dromedary camel swab samples was deduced by sequencing PCR amplicons with overlapping sequence reads. Genome sequence of the virus was constructed by concatenating the amplicon sequences obtained. The sequencing was done with 3–4 times coverage. Genes of the dromedary MERS-CoV was identified by ORF prediction and with reference to published human MERS-CoV genomes. Dromedary MERS-CoV genomes were aligned with human MERS-CoV genome sequences retrieved from

GenBank. Phylogenetic trees were constructed by using MEGA5 with neighbor-joining and bootstrap resampling (*2*).

Virus Isolation

A 200-µL aliquot of original sample was filtered through 0.4-µm filters (Millipore, Billerica, MA, USA) and 100 µL aliquots of neat 1:10 diluted and 1:100 diluted samples were inoculated into Vero E6 cell (ATCC CRL-1586) in T25 flasks with ≈80% confluence. The inoculated cells were kept in minimum essential medium without fetal bovine serum and incubated at 37°C for 6 days. The cells were examined daily to detect virus cytopathic effect (CPE). Upon appearance of CPE, or at day 6 if no CPE was observed, the cells were harvested and passaged for a second time in Vero E6 cells. The cells and supernatant of flasks showing CPE was harvested, aliquoted, and stored at –80°°C.

Serology

HIV pseudoparticles bearing the MERS-CoV spike protein was prepared as described (*3*). HIV/MERS pseudoparticles (5 ng of p24) were pre-incubated with serially diluted heat inactivated serum for 30 min at 4°C and then added to Vero E6 cells (ATCC CRL-1586) in triplicate in 96-well microtiter plates. Residual virus replication was assayed at 2 days post infection as described (*3*). The highest serum dilution giving a 90% reduction of luciferase activity was regarded as the ppNT antibody titer.

References

1. Chu DKW, Poon LLM, Gomaa MM, Shehata MM, Perera RA, Zeid DA, et al. MERS coronaviruses in dromedary camels, Egypt. [Internet]. Emerg Infect Dis. 2014 Jun [cited 2014 Mar 31].

2. Tamura K, Peterson D, Peterson N, Stecher G, Nei M, Kumar S. MEGA5: molecular eevolutionary genetics analysis using maximum likelihood, evolutionary distance, and maximum parsimony methods. Mol Biol Evol. 2011;28:2731–9. PubMed http://dx.doi.org/10.1093/molbev/msr121

3. Perera RA, Wang P, Gomaa MR, El-Shesheny R, Kandeil A, Bagato O, et al. Seroepidemiology for MERS coronavirus using microneutralisation and pseudoparticle virus neutralisation assays reveal a high prevalence of antibody in dromedary camels in Egypt, June 2013. Euro Surveill. 2013;18:20574. PubMed
